# Zingerone [4-(3-Methoxy-4-hydroxyphenyl)-butan-2] Attenuates Lipopolysaccharide-Induced Inflammation and Protects Rats from Sepsis Associated Multi Organ Damage

**DOI:** 10.3390/molecules25215127

**Published:** 2020-11-04

**Authors:** Adil Farooq Wali, Muneeb U Rehman, Mohammad Raish, Mohsin Kazi, Padma G. M. Rao, Osamah Alnemer, Parvaiz Ahmad, Ajaz Ahmad

**Affiliations:** 1 Department of Pharmaceutical Chemistry, RAK College of Pharmaceutical Sciences, RAK Medical and Health Science University, Ras Al Khaimah 11171, UAE; 2 Department of Clinical Pharmacy, College of Pharmacy, King Saud University, Riyadh 11451, Saudi Arabia; mrehman1@ksu.edu.sa; 3 Department of Pharmaceutics, College of Pharmacy, King Saud University, Riyadh 11451, Saudi Arabia; mraish@ksu.edu.sa (M.R.); mkazi@ksu.edu.sa (M.K.); usamah-14@hotmail.co.uk (O.A.); 4 Department of Clinical Pharmacy and Pharmacology, RAK College of Pharmaceutical Sciences, RAK Medical and Health Science University, Ras Al Khaimah 11172, UAE; padma@rakmhsu.ac.ae; 5 Department of Botany and Microbiology, College of Science, King Saud University, Riyadh 11451, Saudi Arabia; parvaizbot@yahoo.com

**Keywords:** zingerone, lipopolysaccharide, inflammation, anti-oxidant, cytokine storm, procalcitonin, histopathology

## Abstract

The present investigation aimed to evaluate the protective effect of Zingerone (ZIN) against lipopolysaccharide-induced oxidative stress, DNA damage, and cytokine storm in rats. For survival study the rats were divided into four groups (n = 10). The control group was treated with normal saline; Group II received an intraperitoneal (i.p) injection (10 mg/kg) of LPS as disease control. Rats in Group III were treated with ZIN 150 mg/kg (p.o) 2 h before LPS challenge and rats in Group IV were given ZIN only. Survival of the rats was monitored up to 96 h post LPS treatment. In another set, the animals were divided into four groups of six rats. Animals in Group I served as normal control and were treated with normal saline. Animals in Group II were treated with lipopolysaccharide (LPS) and served as disease control. Group III animals were treated with ZIN 2 h before LPS challenge. Group IV served as positive control and were treated with ZIN (150 mg/kg orally). The blood samples were collected and used for the analysis of biochemical parameters like alanine transaminase (ALT), alkaline phosphatase (ALP), aspartate transaminase (AST), blood urea nitrogen (BUN), Cr, Urea, lactate dehydrogenase (LDH), albumin, bilirubin (BIL), and total protein. Oxidative stress markers malondialdehyde (MDA), glutathione peroxidase (GSH), myeloperoxidase (MPO), and (DNA damage marker) 8-OHdG levels were measured in different organs. Level of nitric oxide (NO) and inflammatory markers like TNF-α, IL-1ß, IL-1α, IL-2, IL-6, and IL-10 were also quantified in plasma. Procalcitonin (PCT), a sepsis biomarker, was also measured. ZIN treatment had shown significant (*p* < 0.5) restoration of plasma enzymes, antioxidant markers and attenuated plasma proinflammatory cytokines and sepsis biomarker (PCT), thereby preventing the multi-organ and tissue damage in LPS-induced rats also confirmed by histopathological studies of different organs. The protective effect of ZIN may be due to its potent antioxidant potential. Thus ZIN can prevent LPS-induced oxidative stress as well as inflammatory and multi-organ damage in rats when administered to the LPS treated animals.

## 1. Introduction

**Figure 1:**
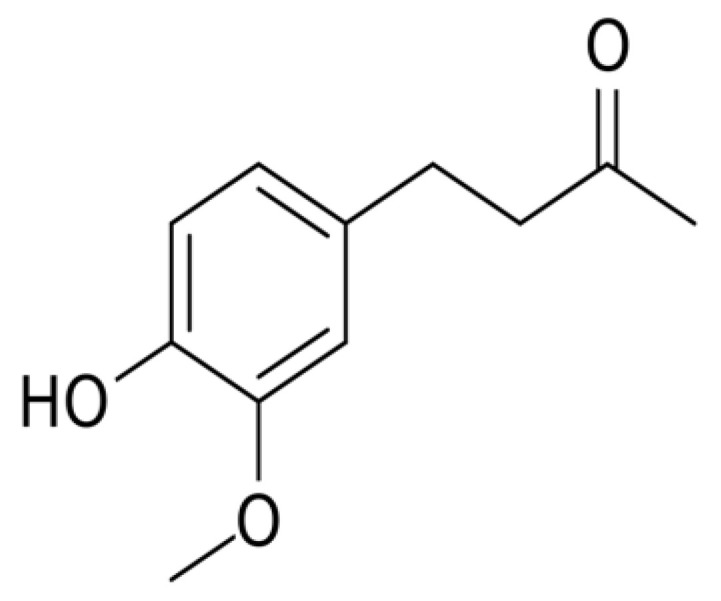
Chemical structure of Zingerone [4-(3-methoxy-4-hydroxyphenyl)-butan-2-one].

Natural compounds have been used as treatments by ancient civilizations in drinks and flavorings. One of the most popular herbs, ginger, is used as a traditional medicine worldwide [1]. Ginger (*Zingiber officinale*) belongs to the family Zingiberaceae. Ginger is a perennial herb used for medicinal and culinary purposes. Ginger is used to treat a variety of ailments around the globe due to its varied phytochemical nature and health benefits [1]. Numerous phytoconstituents are present in ginger which have been divided in to volatile and non-volatile compounds [2]. During drying of ginger, Zingerone is produced directly and by thermal degradation. Zingerone (ZIN) [4-(3-methoxy-4-hydroxyphenyl)-butan-2-one] (Figure 1) is a vanillyl acetone which is a member of phenolic alkanone group [3]. ZIN is present in a significant amount in ginger and it is believed that the pharmacological activities of ginger is due to ZIN. It has a potent antioxidant, anti-inflammatory, anticancer, antidiabetic, antihypertensive, antimicrobial, antithrombotic, anxiolytic, anti-ulcer, and appetite stimulant properties [2].

Lipopolysaccharide (LPS) is a compound that forms part of Gram-negative bacteria, and is a major pathogenic factor in systemic inflammation or sepsis [4]. Gram-negative bacteria are the only species in existence that contain the endotoxic portion of LPS, and lipid. In septic conditions, LPS can activate the innate immunity as a pathogen-associated molecular pattern (PAMP), which mediates an inflammatory response locally or systemically. Also, LPS can activate non-immune cells and start the inflammatory process which is typically detrimental [5,6]. LPS releases inflammatory cytokines in numerous cell types, causing acute inflammatory response towards pathogens [7]. High doses of LPS triggers the release of pro-inflammatory cytokines which causes a detrimental condition known as oxidative stress [8]. Oxidative stress plays a major role in LPS-induced systemic inflammation and is believed to promote the production of reactive oxygen species (ROS). ROS are believed to be involved in the LPS toxicity mechanism [9,10]. The increased ROS level further encourages inflammatory processes and elevate pro-inflammatory cytokines [11]. The first line of ROS mediated inflammation defense mechanism includes multiple antioxidant enzymes, such as: Myeloperoxidase (MPO) and glutathione peroxidase (GSH). Biological compounds (quercetin, resveratrol, curcumin, thymoquinone, and lycopene) with antioxidant properties can help protect against LPS-induced ROS in the tissues and organs [12]. ZIN is one the natural phytoconstituent considered to be reducing oxidative stress due to its potent antioxidant potential [2,13]. Despite of its antioxidant capacity, ZIN can be used to treat these diseases as an efficient medicine. This characteristic owes its potential to scavenge free oxidative radicals. ZIN can therefore suppress ROS and preserve the antioxidant properties. Thus the aim of the present investigation is to study the protective effect of zingerone against LPS-induced oxidative stress, DNA damage, cytokine storm, and histopathological alterations of different organs in rats.

## 2. Results

### 2.1. Effect of ZIN on Survival Rate of Animals

**Figure 2:**
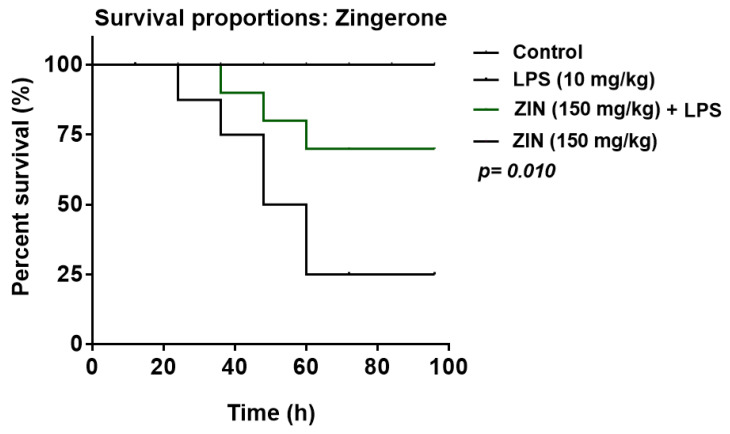
Kaplan–Meier survival plot (n = 10 rats per group). Effect of zingerone (ZIN) 150 mg/kg on survival rate of LPS induce systemic inflammation in rats.

The survival rate of animals was monitored up to 96 h and is illustrated in Figure 2. Zingerone treatment increases the animal survival rate (70%) in lipopolysaccharide (LPS)-challenged animals.

### 2.2. Effect of ZIN on Biochemical Markers

Zingerone treatment exhibit minor changes in the biochemical parameters in the rats as compared with normal rats (Table 1). Animals in the endotoxemia (LPS) group showed significant abnormalities in different organ function markers as apparent by enhanced biochemical marker levels. The hepatic injury markers ALT (145.02 ± 3.84, U/L), ALP (186.54 ± 8.76, U/L) and AST (206.49 ± 11.45, U/L) levels were significantly higher in LPS group as compared to the control group ALT (27.69 ± 3.20, U/L), ALP (64.24 ± 4.32, U/L), and AST (57.65 ± 3.76, U/L), respectively. ZIN treatment decreases hepatic marker levels significantly from ALT (145.02 ± 3.84 to 74.99 ± 6.69 U/L, *p* < 0.05), ALP (186.54 ± 8.76 to 91.29 ± 5.34 U/L, *p* < 0.05) and AST (206.49 ± 11.45 to 87.35 ± 8.29 U/L, *p* < 0.05). General tissue damage of rats was confirmed from serum LDH levels in LPS group, a significantly increase in LDH levels were observed (972.75 ± 20.74 U/L), and these levels were decreased with ZIN treatment (656.59 ± 7.46 U/L *p* < 0.05). Similarly, a reduction in CK (553.83 ± 19.61 U/L *p* < 0.05), SCr (0.38 ± 0.05 mg/dL *p* < 0.05) and, BUN (67.71 ± 2.27 mg/dL *p* < 0.05) were observed in ZIN treated groups as compared to the animals in LPS group CK (855.47 ± 18.84 U/L), SCr (0.59 ± 0.09 mg/dL), and BUN (153.18 ± 7.34 mg/dL), respectively. Also, albumin levels were also increased with the ZIN treatment (3.18 ± 0.013 g/dL *p* < 0.05) as compared to the animals in the LPS group (2.36 ± 0.12 g/dL).

### 2.3. Effect of ZIN on Oxidative Stress and Antioxidant Enzyme Markers

**Figure 3:**
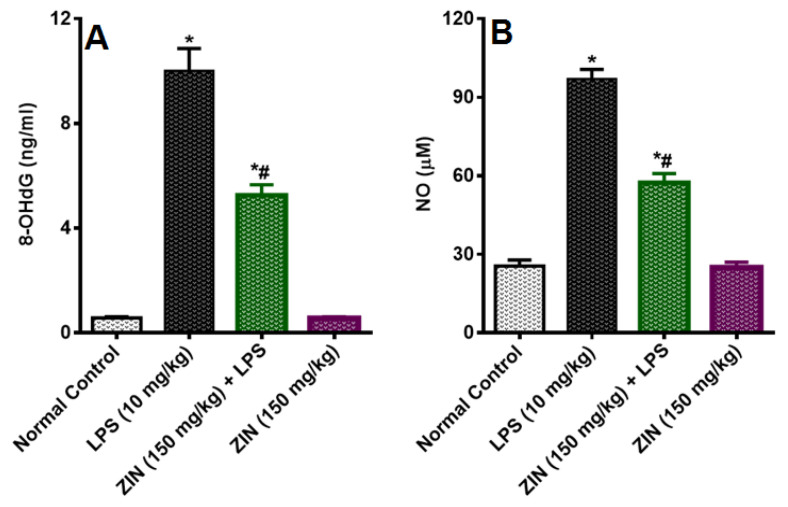
Effect of zingerone (ZIN) on LPS-induced DNA damage and oxidative stress marker. (**A**) 8-OHdG levels (ng/mL), (**B**) Nitric Oxide (NO) levels (µM). Results are represented as mean ± SEM of six rats/group. * *p* < 0.05 vs. control; # *p* < 0.05 vs. LPS group.

**Figure 4:**
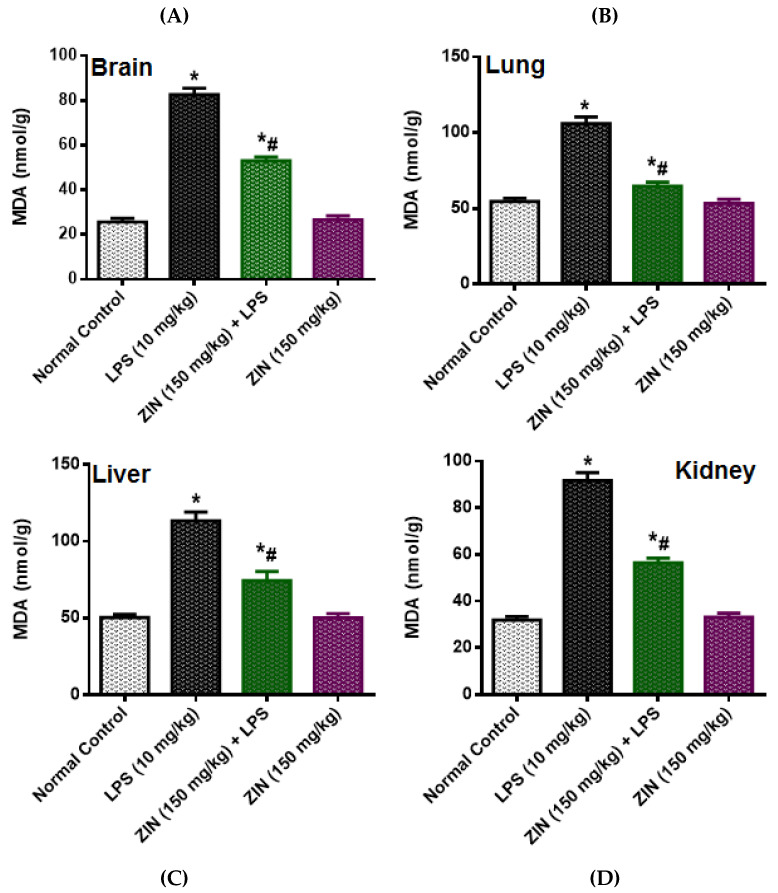
Effect of zingerone (ZIN) on LPS-induced oxidative stress as malondialdehyde (MDA) (nmol/kg). (**A**) Brain, (**B**) lung, (**C**) liver, and (**D**) kidney. Results are represented as mean ± SEM of six rats/group. * *p* < 0.05 vs. control; # *p* < 0.05 vs. LPS group.

**Figure 5:**
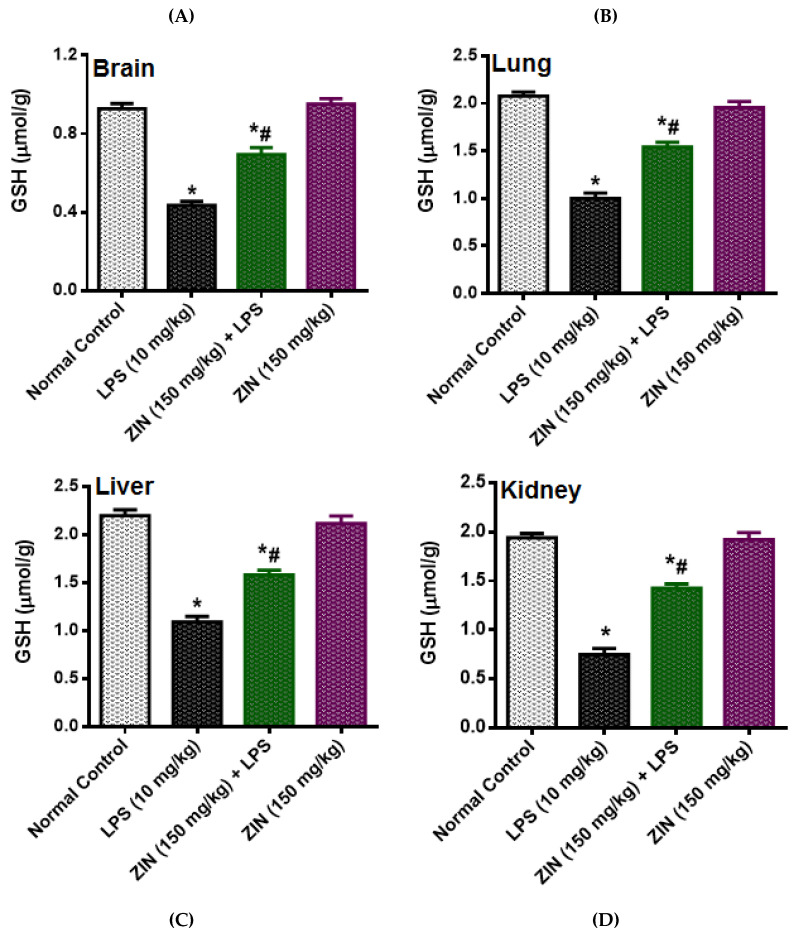
Effect of zingerone (ZIN) on LPS-induced oxidative stress as GSH (µmol/kg). (**A**) Brain, (**B**) Lung, (**C**) Liver and (**D**) Kidney. Results are represented as mean ± SEM of six rats/group. * *p* < 0.05 vs. control; # *p* < 0.05 vs. LPS group.

**Figure 6:**
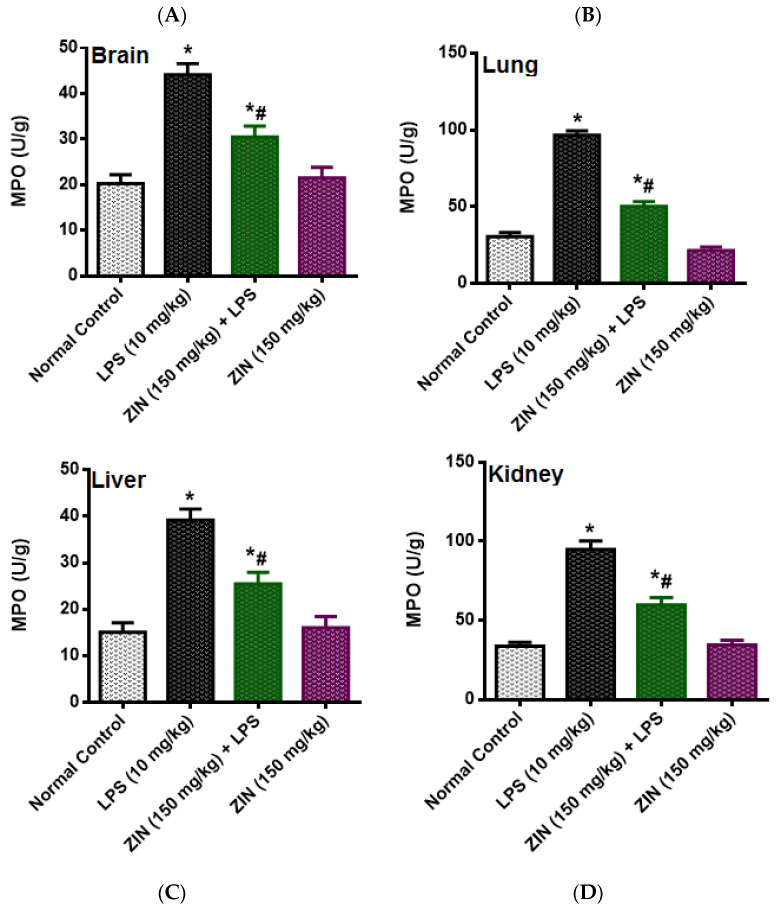
Effect of zingerone (ZIN) on LPS-induced oxidative stress as MPO (U/g). (**A**) Brain, (**B**) lung, (**C**) liver, and (**D**) kidney. Results are represented as mean ± SEM of six rats/group. * *p* < 0.05 vs. control; # *p* < 0.05 vs. LPS group.

The LPS injection elicited levels of NO (97.08 ± 3.64 µM), which was significantly diminished in ZIN treated animals (57.36 ± 3.50 µM *p* < 0.05). 8-OHdG an indicator of DNA damage, the levels were significantly enhanced in the LPS treated groups (10.02 ± 0.84 ng/mL) and this was decreased with ZIN treatment (5.26 ± 0.40 ng/mL *p* < 0.05), respectively (Figure 3). Oxidative stress markers in different tissues e.g., brain, kidney, lung, and liver were measured in terms of glutathione (GSH), malondialdehyde (MDA), and myeloperoxidase (MPO) levels (Figure 4, Figure 5 and Figure 6). MPO an enzyme of activated Polymorphonuclear (PMN) were used as an indication of tissue neutrophil accumulation. ZIN treatment in all tissues showed a significant (*p* < 0.05) reduction of these oxidative markers which was increased in LPS treated animals.

### 2.4. Effect of ZIN on Cytokines and Inflammatory Markers

**Figure 7:**
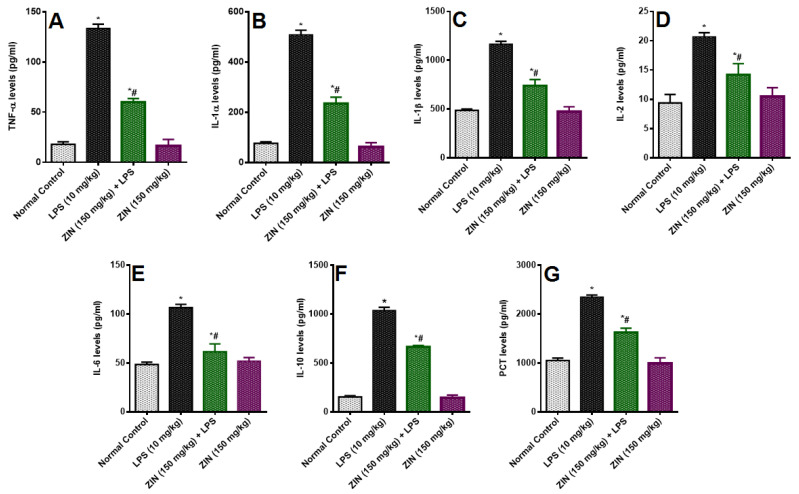
Effect of zingerone (ZIN) on LPS-induced Proinflammatory cytokines and sepsis biomarker PCT (pg/mL). (**A**) TNF-α, (**B**) IL-1α, (**C**) IL-2, (**D**) IL-6, (**E**) IL-8, (**F**) IL-10, and (**G**) procalcitonin (PCT). Results are represented as mean ± SEM of six rats/group. * *p* < 0.05 vs. control; # *p* < 0.05 vs. LPS group.

The inflammatory changes in the LPS-induced animals were determining as their cytokine levels (Figure 7). LPS-induced rats demonstrated a significant increase in the plasma levels of TNF-α (135.56 ± 4.17 pg/mL), IL-1α (509.12 ± 17.79 pg/mL), IL-1ß (1166.01 ± 27.54 pg/mL), IL-2 (20.67 ± 1.70 pg/mL), IL-6 (106.56 ± 3.44 pg/mL) and IL-10 (1037.49 ± 31.65 pg/mL). Zingerone treated animals showed reduced levels of these cytokines as compared to the disease control animals TNF-α (60.16 ± 3.52 pg/mL *p* < 0.05), IL-1α (235.49 ± 10.07 pg/mL *p* < 0.05), IL-1ß (739.78 ± 25.57 pg/mL *p* < 0.05), IL-2 (14.19 ± 1.77 pg/mL *p* < 0.05), IL-6 (61.60 ± 3.21 pg/mL *p* < 0.05) and IL-10 (665.90 ± 14.17 pg/mL *p* < 0.05). PCT levels were determined in the plasma of rats and were significantly increased in the LPS treated animals (2351.65 ± 39.75 pg/mL *p* < 0.05) compared to the normal rats (1053.03 ± 49.49 pg/mL). ZIN treatment significantly diminish the plasma PCT levels (1626.83 ± 86.70 pg/mL *p* < 0.05) Figure 7G.

### 2.5. Effect of ZIN on Histoarchitecture of Different Organs

**Figure 8:**
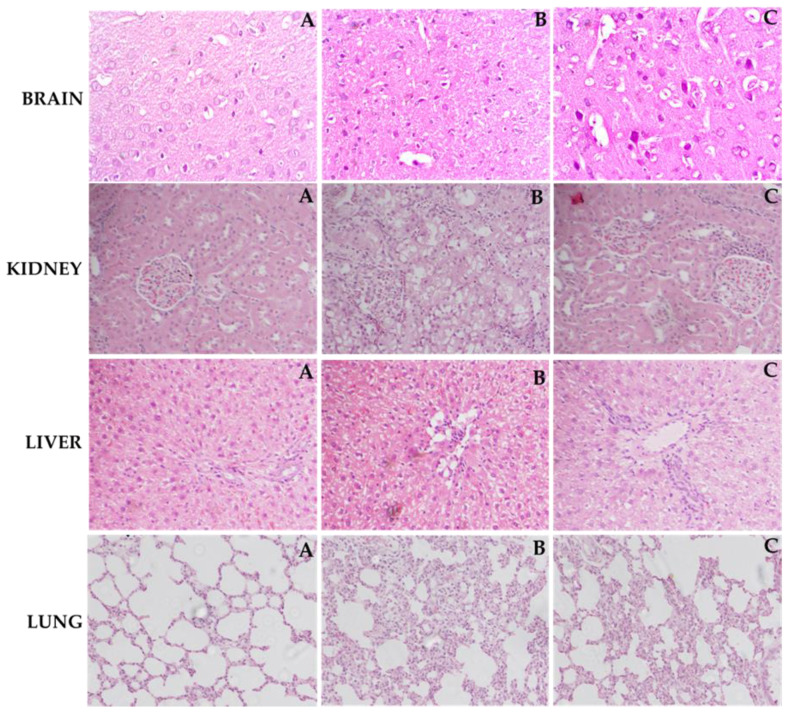
Light histograms of different rat organs (hematoxylin and eosin stains, magnification 40× and scale bar of 100 µm). Effect of ZIN in LPS intoxicated rats. Brain (**A**) normal control group: Showing normal histological structure with intact neurons, (**B**) LPS treated rats: Showing neuronal loss with condensed nuclei, (**C**) Rats treated with (ZIN 150 mg/kg + LPS: Showing decrease in neuronal loss with presence of intact neurons. Kidney (**A**) normal control group: Showing normal histological structure of the glomeruli and tubules at the cortex with absence of histopathological alterations, (**B**) LPS treated rats: Showing marked inflammatory cell aggregation in between the tubules, marked degeneration in the lining epithelium of all the tubules, and blood vessel congestion, (**C**) Rats treated with (ZIN 150 mg/kg + LPS: Showing absence of histopathological alterations. Liver (**A**) Normal control group: Showing normal histological structure of the central vein and intact hepatocytes, (**B**) LPS treated rats: Showing severe loss of hepatic architecture with multiple focal necrosis, ballooning degeneration in the hepatocytes, (**C**) Rats treated with ZIN 150 mg/kg + LPS: Showing absence of histopathological alterations. Lung (**A**) Normal control group showing normal morphology, (**B**) LPS treated rats: Showing moderate to severe hemorrhage, thickening of alveolar septa, emphysema, and infiltration of leukocytes in walls alveoli, (**C**) Rats treated with ZIN 150 mg/kg + LPS: Showing absence of histopathological changes.

As depicted in Figure 8, the tissue sections from normal control group of different organs (brain, lung, liver, and kidney) exhibit normal architecture. Brain, lung, liver, and kidney tissues from LPS group exhibited the pathological alterations comprising widespread inflammation, portal inflammation, and hepatic cell necrosis, infiltration of inflammatory cells, severe hemorrhage, and thickening of alveolar septa, emphysema, and infiltration of leukocytes in walls alveoli and neuronal loss and condensed nucleus. However, treatment with ZIN 150 mg/kg significantly prevented the LPS-induced pathological changes and restored the histological architecture.

## 3. Discussion

LPS is a significant component of Gram-negative bacteria’s outer membrane and can induce inflammatory reactions by penetrating into the bloodstream that can trigger pain and eventually to death [14]. In the current investigation LPS caused impairment of kidney and liver function markers used for the determination of respective organ injuries. This increase in biochemical markers by LPS has been previously reported [15]. LPS has been suggested to generate NO and other ROS which causes peroxidation and cell alterations which further leads to the disruption of cell membrane and subsequent release of cytosolic contents [16]. Moreover, albumin levels were decreased in the in the present investigation which are corroborated with the reports of Amin et al., 2020 [12]. Bilirubin levels in LPS-induced animals were increase 4 fold compared to the normal control animals. ZIN treatment markedly mitigated the alterations induced by LPS by diminishing the levels of CK, Scr, alanine transaminase (ALT), alkaline phosphatase (ALP), aspartate transaminase (AST), blood urea nitrogen (BUN), and bilirubin (BIL) and increasing levels of albumin which is supported by the previous results of Amin et al., 2020 [15].

LPS treatment causes increase in the circulating nitrite levels in LPS intoxicated rats as compare to normal control animals. It is well known that LPS causes’ significant increase in the nitrite levels because of over expression of inducible nitric oxide (iNOS) [17]. The endothelial cells are responsible for the increasing circulating levels of NO at the site of infection in response to pathogen [18]. NO is producing a potent oxidant peroxynitrite (ONOO^−^) by reacting with superoxide anion (O2^−^) which increases the lipid peroxidation and causes oxidative damages in different tissues [19]. The ZIN treatment was found to have protective effect against LPS-induced inflammation as evident by the significant decrease in circulating NO levels. These results were in agreement with the results of Alkharfy and colleagues in 2015, reported that thymoquinone significantly decrease the plasma levels of nitric oxide and the survival in animal model of sepsis [20]. Oxidative stress is characterized as an imbalance between free radical development and antioxidant defense. Superoxide (02^−^), hydroxyl (OH^−^) and nitric oxide (NO^−^) are among the most common reactive oxygen species. Antioxidants immediately reverse oxidative stress by enzymes, such as GSH, and MPO as well as by plant derived flavonoids [21], GSH (tripeptide thiol), found in cells is the most important anti-oxidant molecule and serves as a protecting agent against pathogen induced ROS and RNS [22] and regulates the cell proliferation, and apoptosis [23]. GSH depletion is an important pathological event in many tissues [24]. MPO is an enzyme located predominantly in the primary neutrophil granules and its key function is to destroy the pathogens, although in some circumstances it yields high amount of oxidants which further leads to tissue damage [25]. ROS destroys the cell membrane, DNA damage to cells, causing oxidation of lipid bilayer releasing MDA as the end product [26,27]. MDA, a lipid peroxidation marker used to evaluate the lipid peroxidation due to oxidative stress [28]. ZIN has an exceptional ability of scavenging reactive oxygen species (ROS), free radicals and other damaging oxidants by inhibiting the enzyme xanthine oxidase [13]. Zingerone has also know to exert beneficial effect and protects DNA damage against stannous chloride induced oxidative damage [13]. Zingerone administration has been shown to suppress the mitochondrial injury and lipid peroxidation and mitigates proapoptotic proteins like BAX, and caspases [29]. These findings suggest that zingerone is a potent antioxidant, which was further supported by the fact that ZIN had shown high antioxidant potential as compared to the ascorbic acid [13]. ZIN has shown antioxidant activity against superoxide and NO generated peroxynitrite causing damage to the cells [30]. The plasma levels of these oxidative markers were markedly increased in LPS-induced rats and were significantly reduced with ZIN treatment, suggesting the anti-oxidant potential of ZIN [2,15]. 8-OHdG is one of the principal forms of free radical-induced DNA damage by oxidation, thus been extensively used as an oxidative stress DNA biomarker [31,32]. The increased levels of 8-OHdG were suggested that DNA oxidation was aggravated by LPS administration. ZIN treatment has been found to prevent the LPS-induced oxidative DNA damages as evident by the decreased levels of 8-OHdG.

Elevated cytokines are recognized as inflammatory biomarkers in endotoxin-related pathogenesis [33,34]. TNF-α, IL-β, IL-α, IL-2, IL-6, and IL-10 are known as the key mediators of inflammation and TNF-α among them is considered to be a principal cytokine regulator [35,36]. Many research studies have identified the function of TNF-α and IL-1 in systemic inflammation, including both animal models of septic shock and in human sepsis trials [37,38]. Once released both TNF-α and IL-1 target on different cells e.g., macrophages, neutrophils and endothelial cells. TNF-α contributed to improved macrophage development which stimulates macrophage activation, differentiation and survival of these cells and thus enhances proinflammatory responses in infection [39]. TNF-α and IL-1 enhance inflammatory cascade by activating macrophages to release certain proinflammatory cytokines such as IL-6 and IL-8, ROS/RNS, and lipid mediators which are essential to sepsis-induced organ failure [40]. Increased IL-2 plasma levels with gram-negative bacteria can serve as a septic shock prognostic catalogue. IL-2 receptors are often released from T and B lymphocytes in biological fluids and tend to contribute to sepsis pathogensis. Inflammatory response of IL-6 is likely to be an effective mediator [41]. Endogenous IL-10 is an important anti-inflammatory cytokine, prevents animals from death in sepsis and has been recognized as a key approach for dealing several inflammatory disorders [42]. IL-10 has been reported to be beneficial in various inflammatory diseases other than sepsis or systemic inflammation such as inflammatory bowel disorder, arthritis, sclerosis [41]. In the present study, ZIN treatment to LPS treated rats showed the marked reduction in plasma cytokine levels significantly as compared to LPS treated rats. It has also been observed that the treatment with ZIN in normal control rats does not alter the physiological state. PCT is deemed one of the best biomarkers for sepsis and endotoxemia [43]. LPS toxicity is followed by its binding to lipopolysaccharide binding protein (LBP), which facilitates binding to the CD14 co-receptor, activating cell responses through TLR4 signaling. Reducing the circulating endotoxin in animals handled with ZIN indicates this flavonoid has the potential to enhance LPS clearance. Endotoxin removal occurs in the liver Kupffer and parenchyma cells where it is catabolized. The involvement of ZIN against LPS-induced inflammation and oxidative damage in governing this protective activity further supports this possibility.

LPS-induced rats exhibiting enhanced levels of biochemical parameters were accompanied by enhanced pathological alterations of brain, lung, liver, and kidney. Staining of the brain cortex in the LPS group showed degenerated neurons with hyperchromatic nuclei and increased vacuoles which were prevented by ZIN treatment. The hepatic tissues from the normal control animals exhibited normal cellular and lobular architecture. Liver tissue from the LPS group exhibited prominent pathological alterations comprising widespread portal inflammation, hepatic cell necrosis, and infiltration of inflammatory cells. However, ZIN significantly ameliorated the LPS-induced pathological alterations as demonstrated by the reduced cell infiltration and restored lobular architecture. Kidney tissues from ZIN-treated rats showed gradual but sustained recovery in cortex and medulla structure. Although the recovery is not full, it is easily noticeable in morphology. The thick descending and ascending parts of Henle loops and collecting coils of small caliber and reduction of interstitial tissue can be seen. The tubules show considerably lower cubic epithelium tubules were gaining normal morphology when treated with ZIN. The histopathological evaluation of lung samples indicated a moderate to severe hemorrhage, thickening of alveolar septa, emphysema, and infiltration of leukocytes in alveoli walls in the LPS group. Treatment with ZIN ameliorated these aberrations in the architecture of lung tissues. The improvement in histology is attributed to antioxidant and anti-inflammatory potential of ZIN as suggested by earlier reports [29,44]. Therefore, this research indicates that ZIN, in addition to its antioxidant and anti-inflammatory effects, may reduce the amount of circulating endotoxin from circulation and thereby alleviate the related multi-organ dysfunction syndrome (MODS) when administered to the animals subjected to LPS-induced sepsis.

## 4. Materials and Methods

### 4.1. Chemicals and Reagents

ZIN (99% pure) and LPS were purchased from Sigma-Aldrich. Biochemical estimation kits like ALT, ALP, AST, BUN, Cr, Urea, and CKMB were obtained from Human diagnostics. Oxidative stress markers MDA (K739), GSH (K464), MPO (K747), and (DNA damage marker) 8-OHdG (K4160) were purchased from (BioVision, Inc. Milpitas, CA, USA). Nitric oxide (NO) was obtained from Booster Bio, USA. Tumor necrosis factor-alpha (TNF-α, K1052-100), Interleukin (IL) IL-1β (K4796-100), IL-1α (E4804-100), IL-2 (E4831-100), IL-6 (K4145-100) were procured from (Bio-Vision, Milpitas, CA, USA), IL-10 (MBS355232) and Procalcitonin (PCT, MBS162052), a septic biomarker was purchased from (My BioSource, San Diego, CA, USA). All other chemicals were of analytical grade.

### 4.2. Animals

Six-week-old male Wistar rats (180–200 g) were obtained from an animal facility. The rats were housed in plastic cages at room temperature (25 °C) and humidity of 10% along with 12:12 light–dark cycle. The rats were given free access to water and food. The study was approved by the ethical committee. The study protocol was approved by the Institutional Review Board (No. RAKMHSU-REC-08-2019-F-P).

### 4.3. LPS-Induced Endotoxemia and Survival Study

The rats were divided into four groups (n = 10). The control group was treated with normal saline; Group II received an intraperitoneal (i.p) injection (10 mg/kg) of LPS as disease control. Rats in Group III were treated with ZIN 150 mg/kg (p.o) 2 h before LPS challenge. Animals in Group IV were treated with ZIN 150 mg/kg p.o only. Survival of the rats was monitored up to 96 h post LPS treatment.

### 4.4. Experimental Model

The animals were divided into four groups of six rats in each group. Animals in Group I served as normal control and were treated with normal saline. Animals in Group II served as disease control and IV were treated with LPS (10 mg/kg, i.p). Group III animals were treated with ZIN 150 mg/kg p.o 2 h before LPS challenge. Group IV served as positive control and were treated with ZIN 150 mg/kg p.o.

### 4.5. Determination of Biochemical, Oxidative Stress and Inflammatory Markers

Animals were euthanized with isoflurane after six hours of LPS challenge and blood samples were collected in sterilized EDTA tubes. Different organs like liver, lung, kidney, and brain tissues were harvested. The plasma samples were stored at −80 °C for biochemical, oxidative, and inflammatory marker analyses. Biochemical parameters like ALT, ALP, AST, BUN, Cr, Urea, LDH, Albumin, bilirubin, total protein, and CKMB were assessed. Oxidative stress markers MDA, GSH, MPO, and (DNA damage marker) 8-OHdGlevels were measure by ELISA technique as per the manufacturer details. Levels of nitric oxide (NO) were measured in plasma by Griess reaction method. The color intensity was recorded at 540 nm using ELISA plate reader. Inflammatory markers like TNF-α, IL-1β, IL-1α, IL-2, IL-6, and IL-10 were quantified using ELISA kits as per their manufacturer protocol. The plasma procalcitonin, a septic biomarker was determined using ELISA kits obtained according to the manufacturer’s instructions.

### 4.6. Histological Evaluation

The tissues of liver, lung, kidney, and brain from each animal were extracted and fixed in 12% formalin for histopathological evaluation. The tissues were gradually dehydrated and embedded in paraffin, cut into 4 µm sections, and were stained with Hematoxylin and Eosin for histological examination [45].

### 4.7. Statistical Analysis

Animal survival study was checked using Kaplan–Meier analysis plot. The values are represented as mean ± SEM and the analysis of variance was checked with Dennett’s post hoc test. The analysis was carried out by using GraphPad Prism. *p* < 0.05 was considered significant.

## 5. Conclusions

The present investigation reported the effect of ZIN against LPS-induced oxidative stress, DNA damage, and cytokine storm on various organs by evaluating the biochemical, inflammatory, oxidant-antioxidant markers, and histopathological changes. ZIN in regulating its preventive effect against systemic inflammation further encourages the need to improve it as a more successful treatment. Since several of the novel, creative approaches to treating sepsis target particular biomarkers, better strategies to improve the effectiveness of these new treatment methods would benefit. The results have demonstrated that LPS-induced toxicity and ZIN protects and balances the oxidant-antioxidant status and regulate the generation of cytokine storm. Therefore, ZIN can be beneficial in MODS.

## Figures and Tables

**Table 1:** List of plasma biochemical levels of different groups.

S.No.	Parameters	Control	LPS (10 mg/kg)	ZIN (150 mg/kg) + LPS	ZIN Only (150 mg/kg)
1	Creatinine Kinase (U/L)	68.32 ± 3.92	855.47 ± 18.84 *	553.83 ± 19.61 *#	66.67 ± 3.22
2	Serum Creatinine (mg/dL)	0.30 ± 0.03	0.59 ± 0.09 *	0.38 ± 0.05 *#	0.27 ± 0.01
3	BUN (mg/dL)	42.93 ± 1.92	153.18 ± 7.34 *	67.71 ± 2.27 *#	41.05 ± 0.59
4	LDH (U/L)	307.61 ± 12.27	972.75 ± 20.74 *	656.59 ± 7.46 *#	279.28 ± 7.50
5	ALT (U/L)	27.69 ± 3.20	145.02 ± 3.84	74.99 ± 6.69 *#	30.23 ± 1.43
6	ALP (U/L)	64.24 ± 4.32	186.54 ± 8.76 *	91.29 ± 5.34 *#	61.91 ± 2.65
7	AST (U/L)	57.65 ± 3.76	206.49 ± 11.45 *	87.35 ± 8.29 *#	52.24 ± 3.56
8	BIL (µmol/L)	1.69 ± 0.52	8.55 ± 1.07 *	4.56 ± 0.76*#	1.56 ± 0.75
9	GGT (U/L)	1.34 ± 0.24	3.89 ± 0.65 *	2.16 ± 0.66 *#	1.22 ± 0.34
10	Albumin (g/dL)	3.33 ± 0.12	2.36 ± 0.12 *	3.18 ± 0.013 *#	3.37 ± 0.05

Results are represented as mean ± SEM of six rats/group. * p < 0.05 vs. control; # *p* < 0.05 vs. LPS group. BUN: Blood urea Nitrogen; LDH: Lactate dehydrogenase; ALT: Alanine transaminase; ALP: Alkaline phosphatase; AST: Aspartate transaminase; BIL: Bilirubin; GGT: Gamma-glutamyl transferase.

## Abbreviations

Lipopolysaccharide (LPS); Zingerone (ZIN); Alanine Transaminase (ALT); Alkaline Phosphatase (ALP); Aspartate Transaminase (AST); Gamma-glutamyl transferase (GGT); Blood urea Nitrogen (BUN); Creatinine (Cr); Lactate Dehydrogenase (LDH); Bilirubin (BIL); Malondialdehyde (MDA); Myeloperoxidase (MPO); Glutathione (GSH); Nitric Oxide (NO); Reactive Oxygen Species (ROS); 8-Hydroxy-2′-deoxyguanosine (8-OHdG); Tumor Necrosis Factor (TNF); Interleukins (IL); procalcitonin (PCT).
